# Green Longevity: Climate Resilience, Adaptation, and Action for Older Adults

**DOI:** 10.3389/ijph.2025.1607775

**Published:** 2025-03-10

**Authors:** Chengmeng Zhang

**Affiliations:** Institute of Population Research, Peking University, Beijing, China

**Keywords:** climate action, climate adaptation, longetivity, climate resilience, healthy aging

**The IJPH series “Young Researcher Editorial” is a training project of the Swiss School of Public Health.**

Where climate change and population aging intersect, challenges and opportunities for society arise. While older adults are uniquely vulnerable to the effects of climate change, they can also significantly contribute to climate action and environmental sustainability [1]. “Green Longevity” is a new framework that integrates sustainable green lifestyles with healthy aging. Green longevity highlights the importance of increasing climate resilience in older adults through infrastructure that supports them in adapting their behaviors. The framework aligns with public health strategies that promote environmentally friendly longevity and it establishes the vital role older adults can play in community climate adaptation [2, 3]. Exploring practical strategies for implementing this framework is crucial if we want to promote wellbeing in older adults as the climate is rapidly changing.

Older adults are physiologically, socio-economically, and psychologically vulnerable to climate changes, and so interventions must be comprehensive and targeted. Climate change exacerbates existing health issues, particularly through extreme weather events like heatwaves and cold snaps, which aggravate chronic conditions and raise mortality rates [3]. Climate change also poses risks to the mental wellbeing and social integration of older adults [4]. Many older adults are already isolated, and extreme weather conditions can limit their access to emergency services and community support. Older adults may also suffer psychological stress from climate-related disruptions; for example, displacement or loss from natural disasters disproportionately harms older adults, exacerbating their feelings of isolation and anxiety [5]. Older adults may also be limited by socio-economic constraints that hinder their ability to recover from climate events, deepen their vulnerability and reduce their overall resilience. Interventions must thus be multifaceted approach, encompassing health monitoring, community support, and improving infrastructure [6].

Several promising strategies for increasing climate resilience in older adults warrant our consideration. Among these, urban greening and wellness tourism stand out, since they address both environmental and social needs. Urban green zones have mitigated the effects of urban heat islands. Heat islands disproportionately affect older adults [7] and, along with other urban environmental characteristics, may significantly reduce their mental health [8]. Strategically placing green roofs and community gardens to reduce urban heat islands may cool the environment and increase accessible areas in which older adults can be more physical activity and interact socially, both of which are vital to mental and physical [5]. Wellness tourism is now a burgeoning market in many areas of the world [9]. While developing specialized wellness facilities for climate tourists and integrating green spaces integrated into the design could address health needs of older adults, may benefit some older climate-migrants seeking climate refuge. However, this solution would be complicated to fairly implement, such services are not affordable for all older adults, and may lead to increased housing costs and reduced access to services for older residents in climate-desirable locations.

Integrated solutions can make urban areas more livable for older adults and they align with broader public health goals to support aging populations in locations vulnerable to climate change. A complementary strategy is to engage older adults in both individual and collective climate action. In the Anthropocene where human demands shape emissions patterns, aging is transforming economic structures and consumption patterns. While industrial activities serving human needs are recognized as the primary driver of increased carbon emissions, residents across societies at varying development stages have not achieved their common but differentiated responsibilities. Therefore, engaging older adults in climate action, particularly in high-consumption countries where higher contributions are expected, could catalyze broader green transitions while enhancing their own resilience. Climate action extends beyond micro-level practices like using energy-efficient appliances, prioritizing public transport or walking, and supporting local, sustainable food sources. Collective climate actions by older adults are crucial, as they often lack voice in climate response despite their vulnerability. The dual approach of combining supportive environments with active collective engagement, addresses climate vulnerability in older populations by improving their health outcomes and reducing environmental impacts, enabling them to become exemplars of sustainable living while encouraging broader societal shifts toward greener practices.

Green Longevity is a transformative approach that integrates climate resilience into healthy aging, addressing the multidimensional vulnerabilities of older adults while activating their agency in climate action. Climate change’s causality and attribution are key issues in climate litigation, but these are often complex, as demonstrated by the KlimaSeniorinnen case [10]. Governments have an undeniable responsibility to protect vulnerable populations from the impacts of climate change. Additionally, differential impacts (such as those related to gender, socioeconomic status, etc.) should be considered in any approach to Green Longevity, ensuring that strategies are inclusive and equitable. Coordinated efforts—such as age-sensitive environmental initiatives, integrating climate resilience into geriatric care, and encouraging active participation in stewardship—will establish a foundation for achieving sustainable and healthy aging in an era of climate change.

## Data Availability

The original contributions presented in the study are included in the article/supplementary material, further inquiries can be directed to the corresponding author.
